# Linking local microstructure to fracture location in a two-dimensional amorphous solid under isotropic strain†

**DOI:** 10.1039/d4sm00486h

**Published:** 2024-10-22

**Authors:** Max Huisman, Axel Huerre, Saikat Saha, John C. Crocker, Valeria Garbin

**Affiliations:** a Department of Chemical Engineering, Delft University of Technology Delft 2629 HZ The Netherlands v.garbin@tudelft.nl; b Laboratoire Matière et Systèmes Complexes, CNRS UMR 7057, Université Paris Cité Paris France; c Department of Chemical and Biomolecular Engineering, University of Pennsylvania 220 South 33rd Street Philadelphia PA 19104-6393 USA

## Abstract

Brittle fracturing of materials is common in natural and industrial processes over a variety of length scales. Knowledge of individual particle dynamics is vital to obtain deeper insight into the atomistic processes governing crack propagation in such materials, yet it is challenging to obtain these details in experiments. We propose an experimental approach where isotropic dilational strain is applied to a densely packed monolayer of attractive colloidal microspheres, resulting in fracture. Using brightfield microscopy and particle tracking, we examine the microstructural evolution of the monolayer during fracturing. Furthermore, we propose and test a parameter termed Weakness that estimates the likelihood for particles to be on a crack line, based on a quantified representation of the microstructure in combination with a machine learning algorithm. Regions that are more prone to fracture exhibit an increased Weakness value, however the exact location of a crack depends on the nucleation site, which cannot be predicted *a priori*. An analysis of the microstructural features that most contribute to increased Weakness values suggests that local density is more important than orientational order. Our methodology and results provide a basis for further research on microscopic processes during the fracturing process.

## Abstract 2

Machine learning predicts possible crack location in experiments of a fracturing colloid monolayer, shown along with the actual crack path.

## Graphical abstract

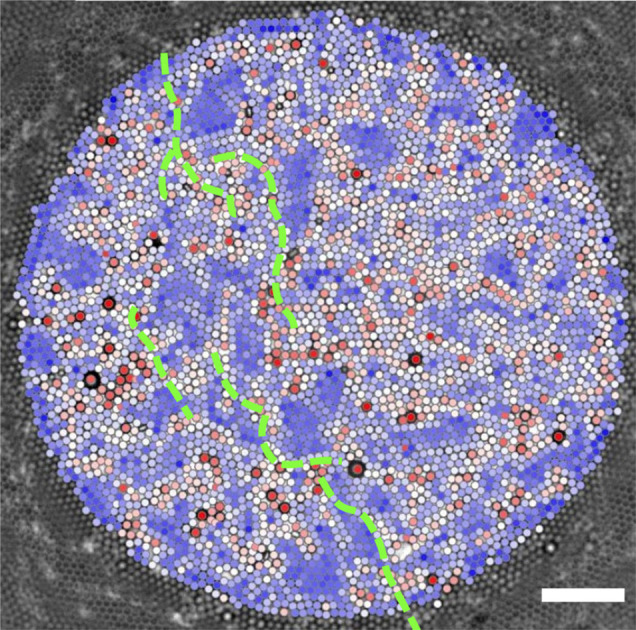

## 1 Introduction

Cracks occur over natural length scales from atoms to earthquakes, but a thorough understanding remains elusive due the unpredictable nature of the fracture process. Generally, materials that display a discontinuous drop in stress at relatively low strain rates, for instance through fracturing, are referred to as brittle. This property is fundamentally different from the continuous evolution over strain of ductile materials.

The field of fracture mechanics was revolutionized by seminal work of A. A. Griffiths,^1^ showing how the decrease of the strain energy by breaking the particle bonds should be higher than the increase in surface energy due to the formation of the free surface during fracturing. These results were generalized to any “somewhat brittle” material in later work,^2^ in which also the main failure modes during fracturing were identified: shear cracks form when stress is applied parallel to the plane of the crack, whereas extensional cracks form when a tensile stress is applied normal to the plane of the crack. Other important early findings show how the stress distribution changes around the propagating crack front.^3,4^ The fracture mechanics theories from these reports use a continuum description of the material, causing the theory to break down near the crack's tip, where the stress field diverges.^5^ Since the microscopic processes occurring in the vicinity of the crack tip are vital in determining the macroscopic process of crack growth and propagation through a material,^6^ it is important to study the dynamics at the small scale.

Recent advancements in simulations and experimental methods have accelerated research into the dynamic material evolution near the crack tip. In simulations, it was shown that cracks tend to initiate in the regions with highest disorder of a brittle amorphous material^7^ and that the direction of crack propagation can be substantially influenced by the presence of defects and voids that lie in front of the crack tip.^5,8^ These findings were confirmed in experiments where the dynamic fracturing of brittle polymeric gels was studied using optical microscopy, showing the important role of defects and voids in crack propagation.^6,9^

Observations from simulations^7,10^ and scattering experiments^11^ strengthen this view by showing how fracturing is governed by localized plastic rearrangements of individual particles, which occur in “soft regions”. Soft regions are regions in a material where particles are most likely to rearrange, characterized by low density and/or high disorder. In the case of attractive particles, particles in soft regions have fewer neighbours that fix their position. Experimental observations on individual particle dynamics in such soft regions during fracturing would be crucial for obtaining a better understanding of the role of microstructure during fracturing, but have to this date not been reported. Individual particle dynamic are often studied using small colloidal particles sized ∼100 nm–10 μm, due to ease of use in combination with various optical microscopy techniques.

Related to fracturing, colloidal systems with small (<100 nm) particles have been used to study macroscale fracturing during drying, relevant to, for instance, the aging of paintings^12^ or dairy stratification.^13^ To allow for live tracking of individual particle movements, colloidal systems with larger particles of size ∼1 μm should be used. An advantage of using a monolayer is the relative ease of imaging all particles in a 2D system with high temporal resolution, and tracking their trajectories using well-established methods.^14,15^ Previously, such experimental systems have been used to study among others the role of defects,^16^ the relaxation time scaling in plastic flow under oscillatory shear,^17^ and the impact propagation through a monolayer after a localized mechanical pulse.^18^

One of the main advantages of individual particle tracking is that one can quantify the microstructure from the particle coordinates over time, through so-called “structural indicators”.^19^ These parameters characterize some important features of the system, such as the local density (for instance through the number of nearest neighbours, or the area of cells in a Voronoi tessellation) or the local order (for instance through orientational order parameters *ψ*_*i*_). Recently, it has been suggested that simple, machine learning (ML) algorithms can also be used to predict how likely individual particles in a sheared system are to undergo plastic rearrangement.^20–22^ The structural indicator called softness characterizes the local structure and was found to be strongly linked to the system dynamics.^20^ An extra incentive for applying ML algorithms to such experimental systems, is that it can be used to identify the most important features in the provided dataset through analyzing the decision making process.

In this paper, we test the extension of such machine learning based methods to experimental systems with macroscopic catastrophic yielding, like fracturing. First, we develop an experimental method where a monolayer of attractive colloids is fractured by applying an isotropic strain. Using brightfield microscopy and particle tracking algorithms we extract particle coordinates, that we use to characterize the monolayer structure and its dynamic evolution. This is done by calculating the orientational bond order parameter and number of nearest neighbours. Since fracture nucleation is a stochastic process, we extend our analyses by using a machine learning method^20–22^ to *a priori* identify regions that are more likely to be on a crack line than others, and we term this structural likelihood the Weakness. Finally, we analyze the relative importance of the microstructural features used as input for the machine learning algorithm, to gain insights into the fracturing process.

## 2 Materials and methods

### 2.1 Sample preparation

We use polystyrene microspheres with negatively charged sulfate functional groups (nominal *d*_avg_ = 5 ± 0.5 μm, ThermoFisher, cat. number: S37227, material lot number: 853189). The colloid suspension (4% w/v) was washed repeatedly by centrifuging and replacing the supernatant with Milli-Q water to remove possible contaminations. The suspension was diluted to 0.4% w/v using a 500 mM NaCl aqueous solution to screen electrostatic repulsion between particles and promote adsorption to the gas–water interface. When the electrostatic interactions are screened, the particles on the interface are attractive. The dominant interaction is capillary attraction by the alignment of quadrupoles in the local interface deformation, and is of the order ∼10^4^*k*_B_*T*.^23^

**Fig. 1:**
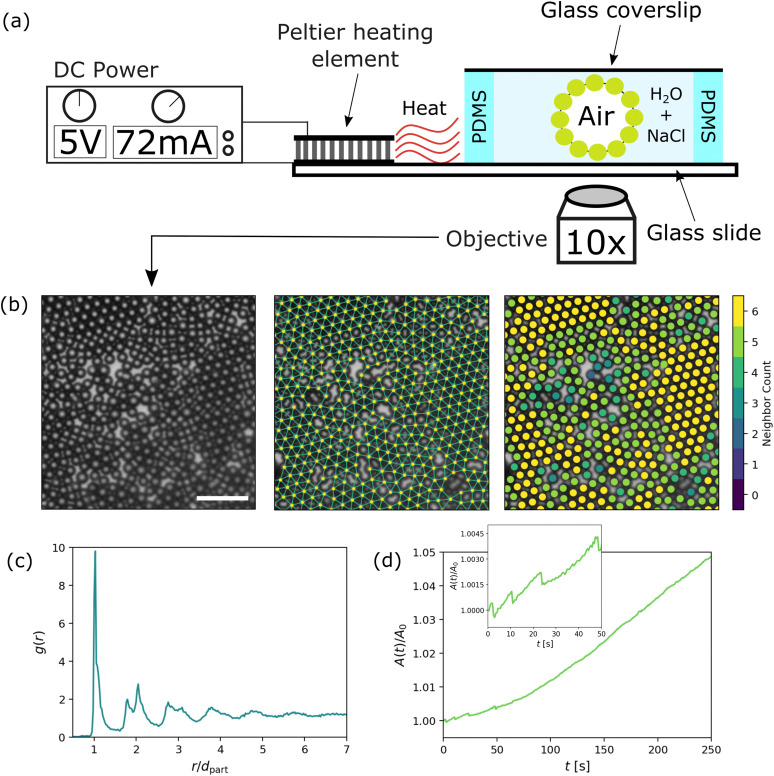
(a) Experimental schematic of the container for a colloid coated air bubble in water. Fracturing is induced through a Peltier heating element connected to DC power, and data is obtained through an optical microscope connected to a camera. (b) A segment of the colloid monolayer, visualized through the raw data, the Delauney triangulation and the amount of nearest neighbours. Scalebar represents 25 μm. (c) Radial distribution function *g*(*r*) for a typical experimental dataset (∼5000 particles). *g*(*r*) is normalized by the first peak value and the radial distance from the central particle *r* is normalized by the measured effective distance between particles *d*_part_ = *r*_*ij*_ = 5.4 μm. (d) Areal expansion (*A*(*t*)) of the perimeter of a colloid coated air bubble in water, normalized by it's initial value *A*_0_. This data was obtained using 5× magnification to track the entire perimeter over time. Inset shows a zoom-in of the initial stages of areal expansion.

To produce colloid-coated air bubbles in water, we thoroughly shake the colloidal suspension to create air bubbles, which also agitates the colloids so that they adsorb at the interfaces of the air bubbles in water. The resulting colloid-coated bubbles are sufficiently stable that they can be individually extracted from the vial using a spatula. The bubble was then placed atop a sample holder, consisting of a 4 mm thick PDMS spacer on a glass slide (76 × 26 mm^2^), filled with a 500 mM NaCl solution and subsequently covered by a glass coverslip (18 × 18 mm^2^). Next, the sample was left undisturbed for at least 10 minutes to equilibrate. A Peltier heating element (RS Peltier Module, 1.6 W, 1.6 A, 7 V, 30 × 30 mm^2^) was glued close to the container, for controlling the temperature of the sample. After preparation, the entire sample container was placed on an inverted microscope (IX71, Olympus) equipped with a camera (Basler ace acA5472-17uc) and a 10× objective. A schematic of the experimental setup is shown in Fig. 1(a).

The in-focus part of the monolayer in the field of view [see Fig. 1(b)] contains ∼5000 particles. The typical surface coverage is 
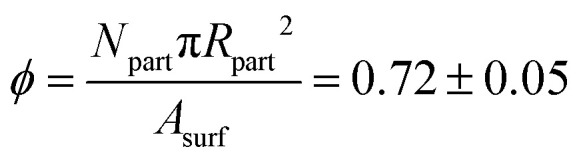
, *N*_part_ being the amount of particles in the field of view, *R*_part_ the particle radius and *A*_surf_ the area of the field of view containing in-focus particles. We note that in our experiments, we found an effective center-to-center distance of *r*_*ij*_ = *R*_part_/2 ≈ 5.4 μm between particles *i* and *j*.

### 2.2 Controlled monolayer expansion and fracture

In previous work, dynamics of colloidal monolayers under strain have for instance been studied through the inflation/deflation of a pendant drop^24^ or by cooling-induced shrinkage of compressible air droplets in water.^25^ Here, we heat colloid coated air bubbles to study extensional fracturing while tracking individual particles, which would otherwise be difficult to study simultaneously. Bubble expansion is achieved by heating the sample holder using a Peltier element. The fluid in the sample holder was heated only by a couple of degrees, ensuring slow expansion to allow for particle tracking.

**Fig. 2:**
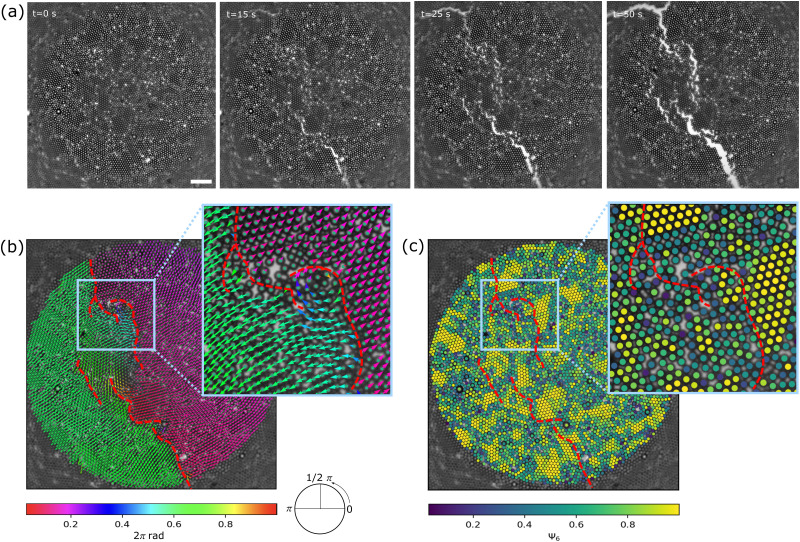
(a) Brightfield images of the colloid monolayer during a typical experiment. Scalebar represents 50 μm. (b) Overlay of drift subtracted displacement vectors on the particle coordinates, comparing the their initial position to their position after a typical experiment (here, *t*_end_ ≈ 100 s). The vectors are coloured by their clockwise orientation, and the red striped lines show where the cracks appear in the system. (c) Overlay of the *ψ*_6_ values on the particle initial particle positions. The red striped lines show where the cracks appear in the system.

The areal expansion of the perimeter of a colloid-coated bubble in a typical experiment is shown in Fig. 1(d). The growth rate slowly increases for *t* < 100 s. During this stage also some sudden drops can be observed (inset of Fig. 1(d)), possibly indicating rapid changes in the structure of the monolayer. After this initial stage (*t* > 100 s), the bubble area increases at a constant rate. We find possible explanations for this behaviour by zooming in at the evolution of a monolayer over time (see Fig. 2(a) and Video S1, ESI†), where we see that the new crack formation mainly occurs in the early stages of bubble growth. These results show a rapid crack propagation through the monolayer that seems heavily influenced by the orientation of the initial crack (more examples of the crack directionality can be observed in Fig. S1 in the ESI†). The rapid changes in the monolayer structure at early times could result in the abrupt changes we observed in the measured perimeter of the bubble in the inset of Fig. 1(d). Next, the bubble proceeds to grow through areal expansion of the already formed cracks, rather than new crack formation, which we assume corresponds to the constant growth rate of the bubble at later times.

### 2.3 Image analysis and particle tracking

To obtain particle coordinates from our microscopy data we used manual particle tracking algorithms in Matlab. We found that a completely automated approach was insufficiently accurate as our microscopy data contains many (∼5000) particles that are subjected to sudden movement, move slightly in and out of focus during an experiment and where the difference between a void and a particle is difficult to detect.

In our approach, we first obtain estimates of initial coordinates using TrackPy.^14^ These were imported to Matlab and refined by manually removing voids classified as particles and adding particles that were not recognised. Even though this procedure was generally robust, there remained some (at least ∼10) misclassified particles in our systems. Particle tracking was performed using the Crocker and Grier algorithm.^15^ When particles experienced a sudden rapid movement or when they moved out of focus such that the tracking algorithm lost a particle, we re-adjusted this particle's position by hand. With an image resolution of 150 nm per pixel, particle tracking has a subpixel accuracy.

To generate sufficiently large datasets to train machine learning algorithms, we performed 20 separate experiments following an identical experimental procedure, which combined together form a dataset containing trajectories of approximately 100 000 particles.

### 2.4 Fracture detection

To quantify the crack location in the monolayer, we identify the particles located on the boundary of a crack. To this end we adapted an image analysis routine originally developed to visualize connectivity in porous media in a time-series.^26^ In our analysis, we used only the grayscale image of the final frame of an experiment. We followed the image processing procedure prescribed in ref. 26 to construct a binary mask from the final frame, showing the largest visible cracks. The cut-off for the crack size was manually adjusted for each experiment. This mask was morphologically dilated by 1 particle diameter and overlaid onto the particle coordinates in the final frame to identify the particles on the edge of a crack. In a typical experiment, a subset of about 100 to 200 particles were located on the edges of cracks out of ∼5000 total particles in the field of view.

### 2.5 Quantifying the local microstructure

A segment of a typical experimental monolayer is shown in Fig. 1(b). We can observe rafts of localized crystalline order, where particles have 6 nearest neighbours (*e.g.* a hexagonal centered packing arrangement), interchanged by more amorphous regions. This observation is in close resemblance to systems from other studies on colloid monolayers with medium range ordering.^17,27,28^ The radial distribution function *g*(*r*) (Fig. 1(c)) confirms this similarity, with local ordering extending up to about 6 coordination shells.

We characterize and investigate the monolayer structure using the number of nearest-neighbours (NN), which relates to the local density, and the bond order parameter *Ψ*_*i*_, relating to the local orientational order. NN is the number of particles within a cut-off radius *R*_c_ = 2 × *r*_part_ (= *r*_*ij*_ = 5.4 μm). We calculate the hexatic bond order parameter as^19^1
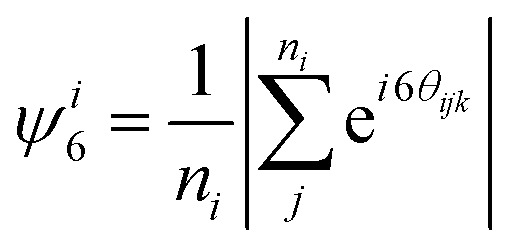
where *i* is the central particle of interest, *θ*_*ijk*_ is the angle of particle *i* with neighbours *j* and *k*. Note that *i* in the exponent is the unit imaginary number. *ψ*^*i*^_6_ is a measure of hexagonal order, with *ψ*^*i*^_6_ → 1 for perfect hexagonal order and *ψ*^*i*^_6_ → 0 otherwise.

### 2.6 A generalized description of local microstructure

We also calculate a structural indicator from a more general description of the local particle environment, using machine learning algorithms. This approach was first proposed by Behler and Parinello^29^ and later applied to the study of plastic rearrangements in colloidal systems.^20–22^

The generalized description consists of two structure functions. The first structure function *G*^*X*^_*Y*_(*i*,*μ*) essentially acts as a discretized radial distribution function, that calculates how many neighbours *j* are located in a shell of thickness *δ* at a distance *μ* from particle *i*, and is defined as2
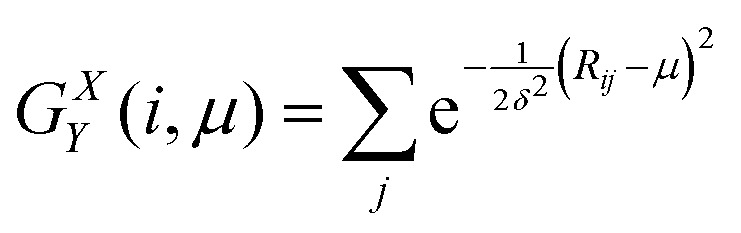
where *R*_*ij*_ is the distance between central particle *i* and neighbour *j*, *δ* is a fixed quantity (in our case *δ* = 0.25 μm), and *μ* is a variable parameter (we used 4.6 μm < *μ* < 12 μm, with steps of 0.1 μm). The cut-off distance from the central particle, *R*_c_, in which this equation is calculated should include several coordination shells but is insensitive to the exact amount.^21^ In total, through varying *μ*, we obtained a set of 75 different values for each particle, which will be referred to as features.

The second structure function *Ψ*^*X*^_*YZ*_(*i*,*ξ*,*λ*,*ζ*), related to orientational properties, is calculated as3
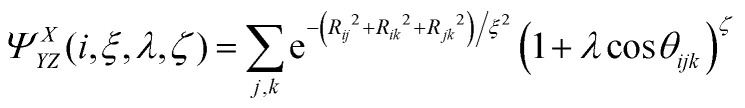


Again, *R*_*ij*_ is the distance between central particle *i* and neighbour *j*, while *θ*_*ijk*_ is the angle that the central particle *i* makes with its neighbours *j*,*k*. *ξ*,*λ*,*ζ* are variable parameters related to different aspects of the particles’ local environment: *ξ* ensures that the Gaussian exponent goes to zero as interparticle distance increases, *λ* (set at either *λ* = 1 or *λ* = −1) determines whether small or large bond angles are used and *ζ* determines or the relative importance of angular properties.^20^ The values we used for the parameters *ξ*,*λ*,*ζ* are given in the ESI,† giving a total of 60 features for every particle.

### 2.7 Calculating Weakness

We want to predict the propensity of a particle to be next to a crack line. To this end, we calculate a parameter that we will refer to as the Weakness, which is a machine learning-generated structural indicator, calculated from the generalized description of the local environment described in Section 2.6. As observed in Fig. 2(a), crack propagation is heavily influenced by the initial crack's directionality. Therefore, we hypothesize that Weakness could identify a likely crack path in the direction of the initial crack, after its formation.

**Fig. 3:**
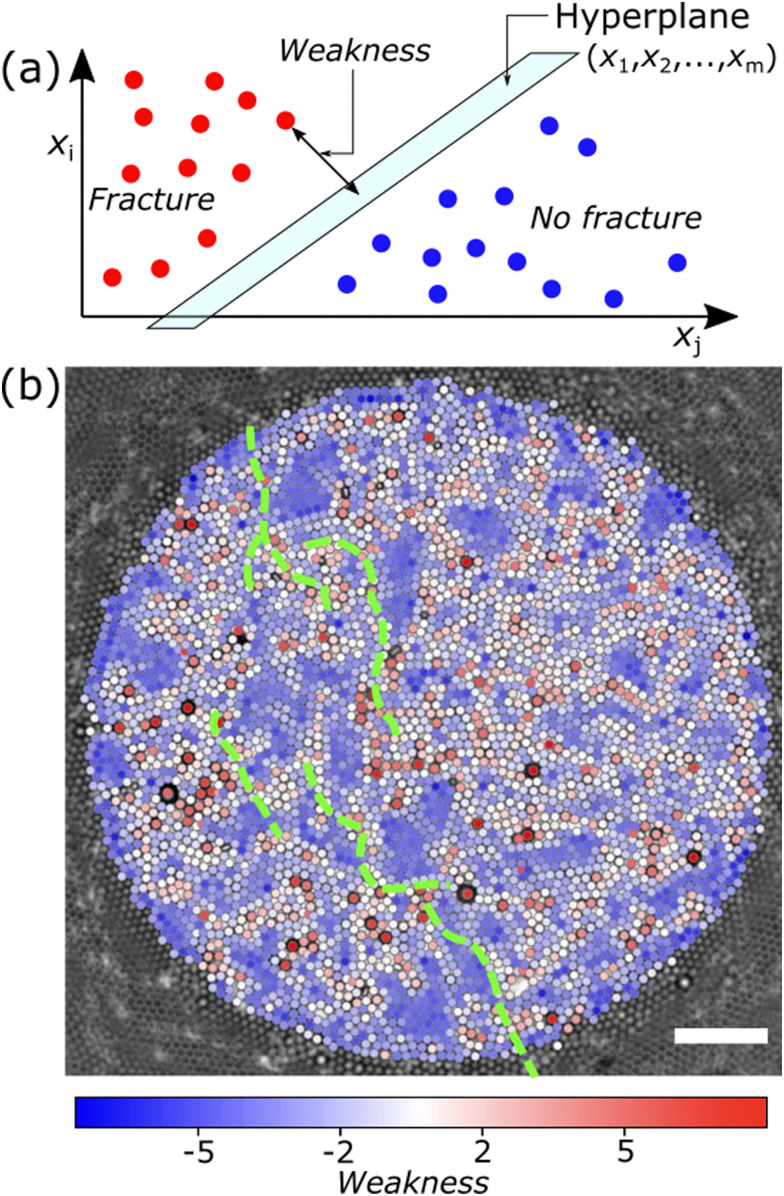
(a) Schematic of the support vector machine (SVM) algorithm. We identify the distance of the particle to the hyperplane as the particle's Weakness. (b) Visual overlay of the calculated Weakness values on the particles in an experimental dataset. Here, particles coloured red are located in a “weaker” local environment, so more prone to fracture, while blue particles are in regions that are less prone to fracture. The approximate location of the fracture in the monolayer is given by the dashed green line. Scalebar represents 50 μm.

As proposed in previous work^20–22^ we employ one of the most straight forward machine learning algorithms: the support vector machine (SVM). Support vector machines (SVM) are supervised classification methods, widely adopted for classification, regression and other learning tasks.^30^ Generally, classification algorithms have a training stage and a testing stage. During the training stage, the SVM takes as input a set of datapoints with features *x*_1_,*x*_2_,…,*x*_*m*_, providing an *m*-dimensional dataset, and for each feature a classification label (0 or 1). In our case, the datapoints are the individual particles and the features are values from eqn (2) and (3). The SVM algorithm then constructs and adjusts a (*m* − 1)-dimensional hyperplane that separates the data into classes 0 and 1 with the highest overall accuracy, see Fig. 3(a). Next, during the testing stage, the hyperplane is fixed and a dataset with datapoints that the algorithm has not seen before, but with the same features *x*_1_,*x*_2_,…,*x*_*m*_, is provided as input. The SVM uses previously calculated hyperplane to predict which of the two outcomes, 0 or 1, is most likely for the new datapoints.

To prevent under- and over-training we optimized the size of our dataset to approximately 12 000 randomly selected particles out of the total 100 000 particles we tracked, see ESI.† The optimal ratio of particles in the dataset was found to be 45% of particles with label 1 (crack) and 55% with label 0 (no crack).

We make use of the simplicity of the SVM to gain insight on the important parameters in the process. This is done by calculating the distance of datapoints from the hyperplane, which is analogous to the probability of the datapoint belonging to its classification class. This distance has been previously used to quantify the probability for plastic rearrangements, and was termed softness in this context.^20–22^ In these reports, the softness values for particles that undergo plastic rearrangements are on average higher compared to other particles, leading to a positive shift in their probability distribution. The distinct separation between the probability distributions in these reports indicates that the hyperplane is able to differentiate the two classes of particles by the values of their structural indicators. Applying a similar method to our experiments, where the prediction labels correspond to the probability of the particle to be located on the edge of a crack, we will refer to this quantity as Weakness.

## 3 Results and discussion

### 3.1 Evolution of the monolayer microstructure during fracturing

Fig. 2(a) and Video S1 (ESI†) show the evolution of the colloid monolayer in a typical experiment. Cracks begin to appear shortly after expansion starts. These cracks propagate through the monolayer until they span the entire field of view, after which crack initiation ceases and crack growth proceeds through areal expansion of the already existing cracks.

With exception of the cracks, the monolayer is not deformed, so that particles move in large rafts with the same magnitude and direction of the particles’ displacement. This is visualized in Fig. 2(b) and inset, which shows clearly the alignment between the directional vectors of the particle movement. After fracturing, particles move away in opposite directions from the crack location, which confirms our system's suitability for studying extensional fracturing dynamics. Also, we observe that some small pockets of about ∼10 particles located on the fracture line sometimes reorient themselves slightly, as seen by the rotational lines in the inset, which is a typical feature in all experiments.

An overlay of the bond order parameter *ψ*_6_ on the particle coordinates is shown in Fig. 2(c). The figure shows that the cracks generally avoid propagation through regions of high *ψ*_6_. This is not unexpected, since domains with *ψ*_6_ → 1 are highly ordered and densely packed so that their constituent particles are mostly surrounded by other particles fixing them in place, in contrast to more disordered domains with low *ψ*_6_ where fewer interparticle bonds have to be broken for a crack to occur, thus requiring less energy.

In systems where particles are similarly sized, regions with low *ψ*_6_ bonds contain voids. Other reports on fracturing suggest that voids in the crack path and near the crack tip are most prone to yielding from crack tip-induced stresses.^7,9^ The crack propagates through the material by rapid growth of these voids and their subsequent coalescence with the main crack. Relating to existing fracture theories would be of great interest to study the stress fields during crack propagation, as for instance shown in ref. 31. However, our particle tracking resolution is not sufficiently accurate to track the very small local particle movement associated with stress buildup. Also it is observed from our experimental data in ESI,† Video S1 that there is no clear propagating crack front: many particles appear to be simultaneously torn apart. Computational methods can be used to provide more insight on this phenomenon.^32^

### 3.2 Identifying weak regions using machine learning

Next, we test ML algorithms for identifying regions that are prone to fracturing, and obtaining a deeper understanding of the fracturing process. First, we show how a simple ML algorithm, the SVM, can identify weak regions in the material. Then, we identify the features of the local particle environments that are most important for determining whether that region is weak.

We show the calculated Weakness value for each particle, obtained using a SVM, for a typical experiment as overlay on particle coordinates in Fig. 3(b). Similar results are obtained for experiments with slightly different surface coverage and average ordering, as shown by the calculated Weakness values in Fig. S1 in the ESI.†

**Fig. 4:**
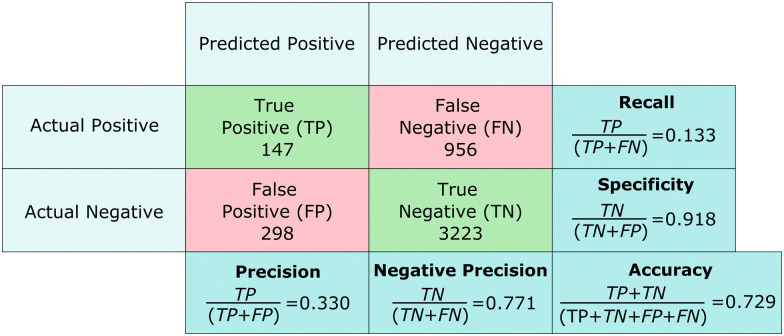
Confusion matrix of the machine learning predictions from the experiment in Fig. 3(b).

We provide the characteristic accuracy indicators of the output of a classification ML algorithm in a confusion matrix in Fig. 4. We compute the overall prediction accuracy Acc_tot_ by dividing the number of correct predictions by the total number of particles, see ESI.† For the experiment of Fig. 3(b), we find Acc_tot_ = 72.9%, with similar results for the other experiments shown in the ESI.† The overall prediction accuracy is however not fully informative, because of the infrequent occurrence of particles participating in a fracture. We also calculate the precision = 33%, which compares the number of particles correctly predicted to be on a crack line (true positives) with the total number of particles predicted to be on a crack line (true positives and false positives), and the recall = 13%, which compares the amount of true positives to the total number of particles that was actually on a crack line (true positives and false negatives). These numbers are both quite low, indicating that the performance of the SVM algorithm in correctly classifying particles that are on a crack line is not good. The negative precision = 77% and the specificity = 91% highlight that the SVM has a high prediction accuracy for predicting that most of the particles are not involved in fracturing, as we also observe from our experimental data in Fig. 3(a).

We do not expect the much more infrequent occurrence of particles on the crack line to have caused a bias during training; to prevent such bias, we used a ratio in our training dataset of 45% of particles with label 1 (crack) and 55% with label 0 (no crack). Rather, we attribute the comparatively low precision and recall in Fig. 4 to the fact that many regions are predicted to be prone to fracture based on their microstructure, but the actual location of a crack depends on nucleation, which is stochastic in nature.

The cracks (green dashed lines in Fig. 3(b)) mostly appear in regions with higher Weakness values, so that Weakness appears to identify a likely crack path in the crack direction. Sometimes the cracks percolate through regions of lower Weakness values, which shows how the direction of the propagating crack can in some cases be dominant over the structural Weakness. We hypothesize that the precise location of the propagating crack is influenced by the interplay between structural Weakness and initial crack direction.

We can characterize the alignment of cracks with regions of high Weakness values by calculating the average Weakness values of particles with label 0 and particles with label 1. We find for particles next to the crack line with label 1, in the experiment in Fig. 3(b), a higher average Weakness value (−0.76) compared to the average Weakness of all other particles (−1.64) with label 0, as shown in Fig. S2 in the ESI.† The distribution of the Weakness values in the figure resembles the observations for softness.^20^ However, the Weakness distribution in our experiments exhibits much greater overlap between the two probability distributions and correlates to the low precision and recall we find for particles with label 1 in Fig. 4. We conclude that the SVM algorithm performs poorly as a classification algorithm for our experimental datasets because it has difficulty distinguishing the two sets of particles with label 0 and label 1 by the values calculated from eqn (2) and (3). This is not unexpected, since we already observed the importance of the directionality and location of the initial crack: the crack direction can be dominant over structural Weakness, meaning that the cracks sometimes propagate through regions with low Weakness values, while the cracks also propagate through only a subset of structurally weak regions in the direction of the initial crack. These observations highlight the importance of stochasticity in our experiments, which is not captured by our input parameters, and makes it unattainable to predict the precise prediction of the crack.

Since our experiments only provide a view of one hemisphere of the colloid monolayer, we are not able to observe crack initiation, which can occur anywhere on the monolayer outside the field of view. We envision how crack initiation in similar experiments can be studied using advanced high-speed 3-dimensional microscopy techniques, which scan the entire size of the armoured bubble (*D* ≈ 4 mm), while tracking the μm-sized particles with a sufficient time resolution.

The observations from the SVM algorithm output agree with the observations from structural indicators like *ψ*_6_: more disordered or lower density domains are more prone to fracturing. This can be seen in Fig. 3(b), where particles in more ordered domains have a negative Weakness value (blue), while particles in more disordered domains are given higher Weakness values (less blue).

The monolayer is slightly polydisperse, and we hypothesize that the larger particles might result in energetically costly point defects in the monolayer, making these points more prone to fracture.^32^ In our experiments, it is possible that the presence of such large particles causes a small out-of-plane displacement, that might affect the capillary interactions between particles and cause dynamic heterogeneity in the system. The rare occurrence of such particles (there are for instance only ∼4 large particles in a typical experiment like Fig. 3(b)), makes a proper analysis of this effect beyond the scope of this paper. Confirming this phenomenology would require experiments with carefully controlled defects, for instance through a controlled variation of the particles’ polydispersity.

### 3.3 Important features in identifying weak regions

We analyze the role of the particle features on the decision-making process of the ML algorithm by investigating more closely the mathematical formulation of the hyperplane in the SVM. The hyperplane location is determined through satisfying the equation ***w***^T^***x***_*i*_ − *b* = 0, where ***w***^T^ is a set of weights for each feature *x*_*i*_, ***x***_*i*_ is a set of all features *x*_*i*_ and *b* is some offset, also referred to as the “bias”. We note here that all our data was normalized to a domain [−1 1] before machine learning. In that case, the values obtained for ***w***^T^ measure the importance of the features in determining the location of the hyperplane.

**Fig. 5:**
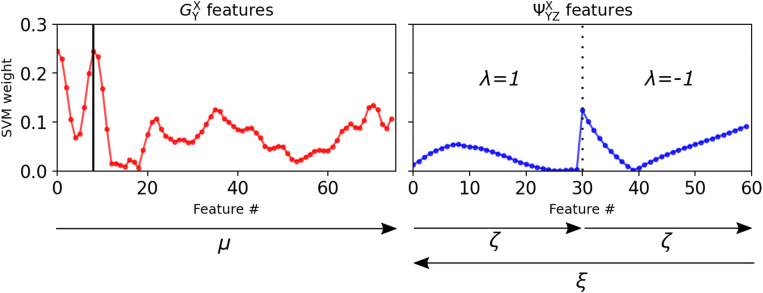
Weights of the SVM features, representing their relative importance. The arrows show the direction and range of the parameter variations, the numerical values are presented in Table S1 of the ESI.† The vertical black line in the *G*^*X*^_*Y*_ plot represents feature number 8, where *μ* = 5.4 μm.

The weights for every feature number, which corresponds to a specific combination of parameters for either *G*^*X*^_*Y*_ or *Ψ*^*X*^_*YZ*_, are shown in Fig. 5. The arrows indicate the range and direction of the varying parameters. The figure shows that the features from the orientation-based *Ψ*^*X*^_*YZ*_ have a lower weight than those from the density-based *G*^*X*^_*Y*_. Features 30 and 60 in *Ψ*^*X*^_*YZ*_ have a relatively high SVM weight because for their parameter combinations the density term e^−(*R*_*ij*_^2^+*R*_*ik*_^2^+*R*_*jk*_^2^)/*ξ*2^ is dominant.

These observations indicate that the local density is more important compared to the local orientational order for determining whether a domain in the material is weak. Even though the features from *Ψ*^*X*^_*YZ*_ also include information on the local density through the term e^−(*R*_*ij*_^2^+*R*_*ik*_^2^+*R*_*jk*_^2^)/*ξ*^2^^ in eqn (3), we still make the conclusion that density is more important, since the addition of angular information in eqn (3) does not in fact lead to higher SVM weight of the orientation-based features.

The profile of the density-based features *G*^*X*^_*Y*_ in Fig. 5 bears resemblance to *g*(*r*), shown in Fig. 1(d). In fact, feature number 8, highlighted by the vertical black line and corresponding to the feature with *μ* = 5.4 μm, we find a peak in the SVM weights. This is striking because *μ* = 5.4 μm corresponds to the same location as the first coordination shell in *g*(*r*). This indicates that the presence, or absence, of particles on the first coordination shell from the central particle is the most important feature in our dataset to determining Weakness.

In Fig. 5 the second coordination shell only corresponds to higher feature numbers approximately between 50 and 70 (where the distance between particles is *r*_*ij*_ ∼ 9.5–11.5 μm). Seeing that there are multiple peaks between these two points, there are most likely recurring configurations of particles that are common in our system, providing information to determining the Weakness of those particles. Future research could make it possible to identify those shapes using shape detection algorithms.

Finally, it should be noted that we also observe high SVM weights at the lowest feature numbers in *G*^*X*^_*Y*_ in Fig. 5. We attribute these to the mis-classification of voids as particles in our experimental system. As explained in Section 2.3, each experimental dataset contained at least order ∼10 misclassified particles, which is probably significant enough to show up in our results. The presence of a (misclassified) void should indeed indicate that there is a void, and thereby lead to a higher propensity to fracture.

## 4 Conclusions

In conclusion, we developed an experimental system to study a fracturing colloid monolayer on a water–air interface under isotropic dilational strain, and used structural indicators and machine learning to obtain more insight into the fracturing process. From brightfield microscopy data we obtained the particle coordinates, from which we quantified the microscructure of the monolayer through structural indicators, for instance *ψ*_6_. These analyses show that cracks tend to propagate through more disordered domains. By defining and calculating the Weakness of domains in the monolayer using machine learning, we show how cracks tend to propagate through regions with slightly increased Weakness values, but not exclusively; crack propagation remains heavily influenced by the stochasticity in the crack's direction and initiation site. This is reflected in the values of the prediction accuracy indicators of the SVM method, which is found to perform poorly as a classifier for our experimental system. Furthermore, in our input parameters, the presence of voids in direct vicinity of the particle was the most important contribution to high Weakness values. Overall, the methodology and results presented here provide a basis for further studies into and understanding of material microstructure during fracturing.

## Data availability

The datasets generated and analyzed in this study are available at the 4TU.ResearchData repository at: https://doi.org/10.4121/c4883858-a901-4e93-b716-2869a664acb0.

## Conflicts of interest

There are no conflicts to declare.

## Supplementary Material

SM-020-D4SM00486H-s001

SM-020-D4SM00486H-s002

## References

[cit1] Griffith A. A., Taylor G. I. (1921). Philos. Trans. R. Soc. London, Ser. A.

[cit2] Irwin G. R. (1957). J. Appl. Mech..

[cit3] Williams M. L. (1961). J. Appl. Mech..

[cit4] Westergaard H. M. (2021). J. Appl. Mech..

[cit5] Rountree C. L., Kalia R. K., Lidorikis E., Nakano A., Van Brutzel L., Vashishta P. (2002). Annu. Rev. Mater. Res..

[cit6] Rozen-Levy L., Kolinski J. M., Cohen G., Fineberg J. (2020). Phys. Rev. Lett..

[cit7] Ozawa M., Berthier L., Biroli G., Tarjus G. (2022). Phys. Rev. Res..

[cit8] Kalia R. K., Nakano A., Omeltchenko A., Tsuruta K., Vashishta P. (1997). Phys. Rev. Lett..

[cit9] Ravi-Chandar K., Knauss W. G. (1984). Int. J. Fract..

[cit10] Pollard J., Fielding S. M. (2022). Phys. Rev. Res..

[cit11] Aime S., Ramos L., Cipelletti L. (2018). Proc. Natl. Acad. Sci. U. S. A..

[cit12] Giorgiutti-Dauphiné F., Pauchard L. (2016). J. Appl. Phys..

[cit13] Floch-Fouéré C. L., Lanotte L., Jeantet R., Pauchard L. (2019). Soft Matter.

[cit14] Allan D. , van der Wel C., Keim N., Caswell T. A., Wieker D., Verweij R., Reid C., Thierry L. G. and Ramos K., apiszcz, zoeith, R. W. Perry, F. Boulogne, P. Sinha, pfigliozzi, N. Bruot, L. Uieda, J. Katins, H. Mary and A. Ahmadia, soft-matter/trackpy: Trackpy v0.4.2, 2019 10.5281/zenodo.3492186

[cit15] Crocker J. C., Grier D. G. (1996). J. Colloid Interface Sci..

[cit16] Buttinoni I., Steinacher M., Spanke H. T., Pokki J., Bahmann S., Nelson B., Foffi G., Isa L. (2017). Phys. Rev. E.

[cit17] Galloway K. L., Ma X., Keim N. C., Jerolmack D. J., Yodh A. G., Arratia P. E. (2020). Proc. Natl. Acad. Sci. U. S. A..

[cit18] Buttinoni I., Cha J., Lin W. H., Job S., Daraio C., Isa L. (2017). Proc. Natl. Acad. Sci. U. S. A..

[cit19] Richard D., Ozawa M., Patinet S., Stanifer E., Shang B., Ridout S. A., Xu B., Zhang G., Morse P. K., Barrat J.-L., Berthier L., Falk M. L., Guan P., Liu A. J., Martens K., Sastry S., Vandembroucq D., Lerner E., Manning M. L. (2020). Phys. Rev. Mater..

[cit20] Schoenholz S. S., Cubuk E. D., Sussman D. M., Kaxiras E., Liu A. J. (2016). Nat. Phys..

[cit21] Cubuk E. D., Schoenholz S. S., Rieser J. M., Malone B. D., Rottler J., Durian D. J., Kaxiras E., Liu A. J. (2015). Phys. Rev. Lett..

[cit22] Sharp T. A., Thomas S. L., Cubuk E. D., Schoenholz S. S., Srolovitz D. J., Liu A. J. (2018). Proc. Natl. Acad. Sci. U. S. A..

[cit23] Huerre A., De Corato M., Garbin V. (2018). Nat. Commun..

[cit24] Huerre A., Cacho-Nerin F., Poulichet V., Udoh C. E., Corato M. D., Garbin V. (2018). Langmuir.

[cit25] Poulichet V., Garbin V. (2015). Langmuir.

[cit26] Mayorga-González R., Rivera-Torrente M., Nikolopoulos N., Bossers K. W., Valadian R., Yus J., Seoane B., Weckhuysen B. M., Meirer F. (2021). Chem. Sci..

[cit27] Schwenke K., Isa L., Del Gado E. (2014). Langmuir.

[cit28] Keim N. C., Arratia P. E. (2014). Phys. Rev. Lett..

[cit29] Behler J., Parrinello M. (2007). Phys. Rev. Lett..

[cit30] Chang C.-C., Lin C.-J. (2011). ACM Trans. Intell. Syst. Technol..

[cit31] Goldman T., Livne A., Fineberg J. (2010). Phys. Rev. Lett..

[cit32] Negria C., Sellerioa A. L., Zapperia S., Miguele M. C. (2015). Proc. Natl. Acad. Sci. U. S. A..

