# Supplementary material for: Linking local microstructure to fracture location in a two-dimensional amorphous solid under isotropic strain
Source: Soft Matter. 2024 Oct 22;20(44):8888–96. doi: 10.1039/d4sm00486h (PMC11525953; doi:10.1039/d4sm00486h)
Supplement: SM-020-D4SM00486H-s002 [file SM-020-D4SM00486H-s002.pdf]

Supplementary Information (SI) for Soft Matter.
This journal is © The Royal Society of Chemistry 2024

Linking local microstructure to fracture location in a two-dimensional amorphous solid under isotropic strain

Supplemental Information

Max Huisman, Axel Huerre, Saikat Saha, John C. Crocker, Valeria Garbin

1 Parameter choices for structure functions

Parameter values for the structure functions $G_Y^X(i, \mu)$ and $\Psi_{YZ}^X(i, \xi, \lambda, \zeta)$, based on the approach used in [1]. Through these parameter variations we obtained 75 features for G and 60 features for Ψ. We manually adjusted these to optimize quantitative output from our SVM and note that, as the paper by Behler and Parinello [2] prescribes, the choice of parameters is not unique but best suits the description of the local environment in this system.

Table 1: Feature values structure functions

**Feature**	**Values**
μ (μm)	4.6, 4.7, 4.8, ... , 11.8, 11.9, 12
ξ(μm)	0.368, 0.243, 0.177, ... , $e^{-\sqrt{\beta}}$, ... , 0.000492, 0.000432 where β=1, 2, 3, ... , 58, 59, 60
ζ	0.1, 0.2, 0.3, ... , 2.9, 3.0, 0.1, 0.2, ... , 2.8, 2.9, 3.0
λ	1, 1, 1, ... , 1, 1, -1, -1, ... , -1, -1, -1

2 SVM optimization

2.1 Choice of kernel and feature importance

In the SVM algorithm, we used a linear kernel for the hyperplane to allow interpretation on the importance of the features.

The hyperplane of the SVM follows the equation $\boldsymbol{w}^{\mathbf{T}}\boldsymbol{x_i} - b = 0$. Here, $\boldsymbol{x_i}$ is a set of all features x_i, $\boldsymbol{w}^{\mathbf{T}}$ is a set of weights for each feature x_i, and b is some offset, also referred to as the "bias". Since $\boldsymbol{w}^{\mathbf{T}}$ provides the weight of a feature x_i to the positioning of the hyperplane, this can be used as a measure of the feature importance of x_i.

We obtain *Weakness* by calculating the distance of datapoints from the hyperplane, which is analogous to the probability of the datapoint belonging to its classification class.

2.2 Dataset size and label distribution

To prevent under- or over-fitting, we calculated the training and testing accuracy for different sized training datasets. The training accuracy was calculated through 5 fold cross validation, and we used a test set of 33,388 particles that were not used during training. The testing and training accuracy are more or less equal in the region around 12,000 datapoints, after which the testing accuracy decreases, which is a sign of overfitting [3]. Thus, the optimal dataset size was 12,000 particles.

We also determined the optimal ratio of particles with label 1 (crack) and with label 0 (no crack). The ratio of the datapoints in the training set was varied from 10:90 to 90:10 [label 1:label 0], in steps of 5%. Considering that equal accuracy for testing and training is optimal [3], we found an optimal ratio 45:55 [label 1:label 0].

3 ML outputs for varying surface coverage

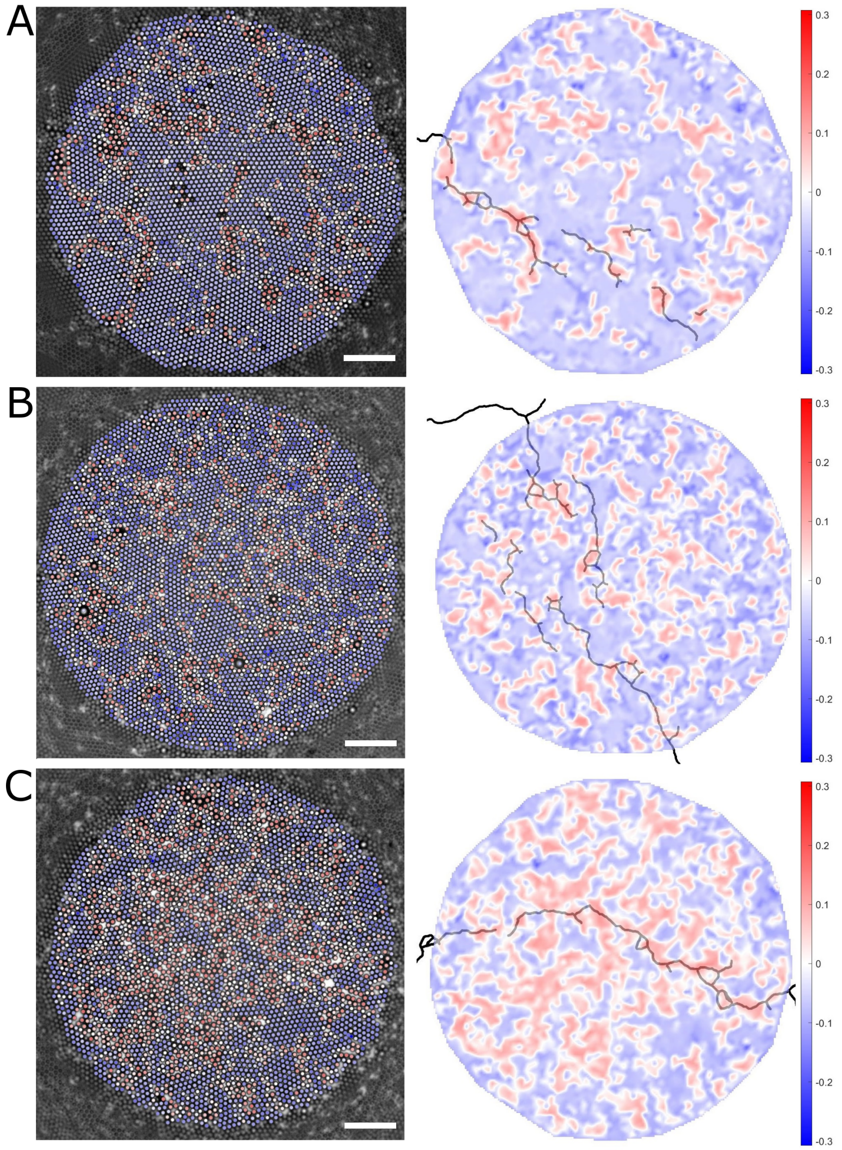

Figure S1: Machine Learning output of an SVM model using physics motivated structural indicators (left), with their corresponding surface map (right) for 3 different samples: **(a)** 3953 particles ($\varphi \approx 0.84$, prediction accuracy = 79.2%) **(b)** 4624 particles ($\varphi \approx 0.72$, prediction accuracy = 74.0%) **(c)** 3562 particles ($\varphi \approx 0.65$, prediction accuracy = 62.6%) The approximate location of the fracture is shown as a black line and the scalebar represent 50 μm.

4 Averages and distributions of Weakness values

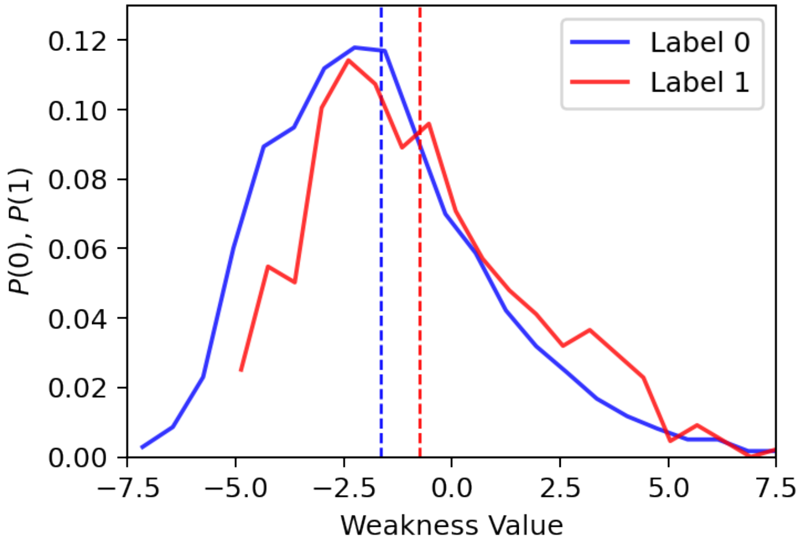

Figure S2: Distribution of predicted Weakness values for the experiment shown in Fig. 3 of the main text. Dashed lines give average Weakness for both distributions.

References

[1] Tristan A. Sharp, Spencer L. Thomas, Ekin D. Cubuk, Samuel S. Schoenholz, David J. Srolovitz, and Andrea J. Liu. Machine learning determination of atomic dynamics at grain boundaries. *Proc. Natl. Acad. Sci. U. S. A.*, 115(43):10943–10947, 2018.

[2] Jörg Behler and Michele Parrinello. Generalized neural-network representation of high-dimensional potential-energy surfaces. *Phys. Rev. Lett.*, 98:146401, Apr 2007.

[3] E. D. Cubuk, S. S. Schoenholz, J. M. Rieser, B. D. Malone, J. Rottler, D. J. Durian, E. Kaxiras, and A. J. Liu. Identifying structural flow defects in disordered solids using machine-learning methods. *Phys. Rev. Lett.*, 114:108001, Mar 2015.
